# Health and the built environment in United States cities: measuring associations using Google Street View-derived indicators of the built environment

**DOI:** 10.1186/s12889-020-8300-1

**Published:** 2020-02-12

**Authors:** Jessica M. Keralis, Mehran Javanmardi, Sahil Khanna, Pallavi Dwivedi, Dina Huang, Tolga Tasdizen, Quynh C. Nguyen

**Affiliations:** 1 Department of Epidemiology and Biostatistics, University of Maryland School of Public Health, 4200 Valley Dr. #2242, College Park, MD 20742 USA; 2 Department of Electrical and Computer Engineering, University of Utah, 50 S Central Campus Dr #2110, Salt Lake City, UT 84112 USA; 3 Master’s in Telecommunications Program, University of Maryland, 2433 A.V. Williams Building, College Park, MD 20742 USA

**Keywords:** Google Street View, Machine learning, Computer vision, Built environment, Structural determinants of health

## Abstract

**Background:**

The built environment is a structural determinant of health and has been shown to influence health expenditures, behaviors, and outcomes. Traditional methods of assessing built environment characteristics are time-consuming and difficult to combine or compare. Google Street View (GSV) images represent a large, publicly available data source that can be used to create indicators of characteristics of the physical environment with machine learning techniques. The aim of this study is to use GSV images to measure the association of built environment features with health-related behaviors and outcomes at the census tract level.

**Methods:**

We used computer vision techniques to derive built environment indicators from approximately 31 million GSV images at 7.8 million intersections. Associations between derived indicators and health-related behaviors and outcomes on the census-tract level were assessed using multivariate regression models, controlling for demographic factors and socioeconomic position. Statistical significance was assessed at the α = 0.05 level.

**Results:**

Single lane roads were associated with increased diabetes and obesity, while non-single-family home buildings were associated with decreased obesity, diabetes and inactivity. Street greenness was associated with decreased prevalence of physical and mental distress, as well as decreased binge drinking, but with increased obesity. Socioeconomic disadvantage was negatively associated with binge drinking prevalence and positively associated with all other health-related behaviors and outcomes.

**Conclusions:**

Structural determinants of health such as the built environment can influence population health. Our study suggests that higher levels of urban development have mixed effects on health and adds further evidence that socioeconomic distress has adverse impacts on multiple physical and mental health outcomes.

## Background

The built environment has long been viewed as a structural determinant of health by social epidemiologists [1]. A substantial body of research has documented the association of built environment characteristics – such as accessibility, physical disorder, access to public transit and recreational spaces, and greenery – with health-related behaviors [2], health outcomes [3–7], and health care expenditures [8]. Features of the built environment may influence health-related behaviors and outcomes through several pathways, including encouraging (or discouraging) exercise and recreational activities, determining whether residents have easy access to healthy foods and preventive health resources, and affecting stress and other psychosocial factors.

Methods to assess the built environment have evolved alongside analyses to measure its association with health. These methods include cross-sectional surveys on residents’ perceptions and observational methods [9]; tax records, land use inventories, and other administrative data sources [4]; and large geospatial data sets developed through satellite imagery, such as the National Land Cover Database [5, 8]. While these have served as valuable resources for creating built environment indicators, it has nonetheless proven challenging to compare or combine analyses because many neighborhood-level data collection initiatives have been specific to the area being studied and thus limited in focus.

Launched in 2007, Google Street View (GSV) is an increasingly popular source of images of the built environment that has the potential to address this gap. GSV is a publicly available source of image data on built environment features which is collected with uniform methodology. It represents a massive volume of detailed data that can be used to create indicators of characteristics of the physical environment with machine learning techniques. This reduces the significant time and resources previously spent on more traditional methods of neighborhood feature data collection, such as in-person audits [10]. Researchers have demonstrated the reliability of using GSV to derive data on physical features, finding high-level agreement with field assessments [11–13]. GSV images have been used to determine the presence of features such as crosswalks, commercial buildings, highways, and grasslands, which were in turn used to assess their association with chronic health outcomes at the county [14] and zip code level [15]. Globally, GSV image coverage is more complete for some regions than others, with cities in developed nations having near-complete coverage while many low- and middle-income countries in Africa, Southeast Asia, and South America have no GSV imagery at all [10]. While precise coverage metrics are not available, the U.S. has near-complete coverage [16, 17].

The aim of this study is to use GSV images, in conjunction with data on demographic and socioeconomic covariates, to measure the association of built environment features with health-related behaviors and outcomes at the census tract level.

## Methods

### Data sources

#### Google street view data for built environment indicators

Indicators for building type (the presence of any building that was not a single-family detached house), single-lane road (yes/no), presence of a crosswalk (yes/no), street greenness (street trees and street landscaping comprised at least 30% of the image - yes/no), and visible utility wires overhead (yes/no) were derived from approximately 31 million GSV images at 7.8 million intersections. The indicators were selected through an iterative process of considering what the literature has found to be important built environment characteristics and what is feasible for computer vision models. Neighborhood walkability [18–20], neighborhood disorder [21–23], and mixed land use [24–26] have been identified in the literature as being important for health outcomes.

The presence of crosswalks is a classic indicator of walkability and was included to measure its influence on health behaviors and related health outcomes. While we also examined sidewalks, in urban areas, the prevalence of sidewalks is high and thus there is less variability with this indicator.

The impact of mixed land use on travel behavior is well-studied. Areas that are single-use residential often lead individuals to use motorized transport to get to destinations. Conversely, areas that blend a mixture of residential, commercial and leisure destinations might allow individuals to walk or bike [27] and be related to greater access to resources, physical activity and better health. We operationalized mixed land use such that labeling images was feasible for both humans (human coders manually labeled images to provide training data to the computer vision models) and machines. Computer vision models struggle with indicators that are too common or too rare (e.g., prevalence of 90% or conversely 10%) because models can merely label all images as having the absence or presence of an indicator and be right most of the time. While looking through images, we noticed that an image could be classified as having only homes or a blend of homes and other building types. Thus, non-single-family home was created to distinguish between purely residential places and places with different building types. The prevalence of this indicator with a median value of around 30% nationally suited the capacity of computer vision models.

We operationalized street greenness as street trees and street landscaping comprising at least 30% of the image. A cut-point of approximately 30% was utilized to assist with inter-rater reliability in manual annotations of street greenness. Moreover, we found that most images had some street landscaping and aimed to create a neighborhood indicator to distinguish between ample versus sparse street landscaping.

From images, we also extracted the presence of visible wires. The literature on visible wires is nascent and more of this work has been done abroad, for instance in Rio de Janeiro, where the wires represent both an unsightly presence and a possible electrocution/electrical fire risk [28]. In the United States, visible wires have mainly a visual impact on the landscape. We chose this indicator to further the literature and to investigate whether visible wires as an indicator of physical disorder might have links to important health outcomes. Other neighborhood indicators of physical disorder were considered, such as litter or trash. However, we found that computer vision models struggled with small objects. In addition, these objects were also difficult to label by humans as well (low inter-rater reliability). Thus, while litter is a classic built environment feature for neighborhood disorder, we could not include this indicator.

Methods for identifying street intersections and retrieving and labeling GSV images have been published previously [14, 15]. Briefly, latitude and longitude data coordinates for all U.S. street intersections were obtained from the 2017 Census Topologically Integrated Geographic Encoding and Referencing (TIGER) data. Intersections were identified with the PostgreSQL (an open-sourceobject-relational database system) with the PostGIS plugin [29]. GSV images of the intersections were then retrieved via Google’s Street View Image Application Programming Interface (API) using these coordinates. For each pair of coordinates corresponding with an intersection, four images (with the camera facing north, east, south, and west) were obtained to capture a 360-degree view of the environment. Image resolution was 640 × 640 pixels. Images were processed using trained Visual Geometry Group (VGG-16 model) deep convolutional networks [30, 31] (previously detailed by Nguyen et al. [15]) to identify the five built environment features of interest (one network per feature). Accuracy of the recognition tasks (comparing the images labeled using this machine learning approach compared with assessment by a human reviewer) ranged from 85 to 93%, and these figures were consistent with a separate, semi-supervised learning approach.

Census tracts are small, relatively permanent statistical subdivisions of a county or equivalent entity, roughly equivalent to a neighborhood. They are established by the U.S. Census Bureau to provide a stable set of geographic units for the presentation of statistical data. Census tracts generally have a population size between 1200 and 8000 people, with an optimum size of 4000 people [32]. The image values of built environment indicators were then aggregated to produce small-area summaries at the census tract level. Each census tract was given an aggregate score ranging from 0 to 1, representing the percentage of GSV images in which the feature was detected. For example, if 50% of the GSV images for a given census tract contained visible wires, that tract was assigned a score of 0.5 for the visible wire indicator.

#### 500 Cities data for health outcomes

Data on census tract-level health outcomes were obtained from the 500 Cities Project, a partnership between the Centers for Disease Control and Prevention (CDC), the Robert Wood Johnson Foundation, and the CDC Foundation [33]. The data contain information on chronic disease measures, including health outcomes, public health prevention metrics, and health-related behaviors, on 500 cities and approximately 28,000 census tracts. Estimates are derived from the Behavioral Risk Factor Surveillance System (BRFSS), Census Bureau 2010 census population data, and American Community Survey (ACS) five-year estimates, and are calculated using small-area estimation methods. Behaviors and outcomes assessed include obesity, diabetes, frequent physical distress, frequent mental distress, physical inactivity and binge drinking. We hypothesized that non-single-family homes, crosswalks, and street greenness would be associated with decreased prevalence of all outcomes. Conversely, single-lane roads and visible wires would be associated with increased prevalence of all health-related outcomes.

#### American Community Survey data for demographic and socioeconomic characteristics

Census tract-level information on demographics and socioeconomic position were included in the analysis to adjust for potential confounding of the relationship between the built environment and health-related behaviors and outcomes. Data on covariates were derived from ACS 2013 5-year estimates. Demographic covariates included median age, percent under age 18 and over age 65, percent white, percent of Hispanic ethnicity, and percent female. To control for socioeconomic position, we used a composite economic factor for socioeconomic disadvantage derived from percent single-parent households, unemployment level, percentage of families living in poverty, high school graduation rate, and percent of residents with some college education. The composite factor was created by conducting a factor analysis of these five variables, using varimax rotation, and taking the first factor. We used a similar approach in previous GSV analyses [14, 15]. We hypothesized that socioeconomic disadvantage would be associated with increased prevalence in these outcomes.

### Analytic approach

To allow for nonlinearities in the association between built environment characteristics and health and to ease presentation of study results, built environment indicators were grouped into high, moderate, and low tertiles, with one third of the census tracts grouped into each tertile for each indicator. Health outcomes were modeled as continuous variables. Adjusted linear regression models were used to estimate differences in the prevalence of the selected health outcomes by tertile of each built environment indicator, using the lowest tertile as the reference group. Models were fit for outcomes and built environment indicators first, then adding for demographic characteristics, and finally including both demographics, median income, and the composite economic factor for socioeconomic disadvantage. Each health outcome was modeled separately. Statistical significance for differences between tertiles was assessed at the α = 0.05 level. Analyses were conducted using Stata IC15 (StataCorp LP, College Station, TX).

## Results

Health outcomes were modeled for 20,121 census tracts with complete data on health outcomes and GSV-derived built environment indicators, representing 416 cities in all 50 states and the District of Columbia. Approximately half of the census tracts were in 40 cities, and two-thirds were in 95 cities. The cities and states with the largest number of census tracts can be seen in Table 1.

**Table 1:** States and cities with the largest number of census tracts

State	Census tracts	Percent	State	City	Census tracts	Percent
California	4162	20.68%	New York	New York	1808	8.99%
Texas	2269	11.28%	California	Los Angeles	933	4.64%
New York	1996	9.92%	Illinois	Chicago	718	3.57%
Illinois	1073	5.33%	Texas	Houston	584	2.90%
Florida	1010	5.02%	Pennsylvania	Philadelphia	381	1.89%
Michigan	651	3.24%	Texas	San Antonio	326	1.62%
Pennsylvania	614	3.05%	California	San Diego	290	1.44%
North Carolina	557	2.77%	Texas	Dallas	279	1.39%
Colorado	556	2.76%	Michigan	Detroit	268	1.33%
Washington	506	2.51%	Hawai’i	Honolulu	236	1.17%
Ohio	426	2.12%	Wisconsin	Milwaukee	208	1.03%
Georgia	395	1.96%	California	San Jose	206	1.02%
Massachusetts	394	1.96%	Texas	Austin	204	1.01%
Tennessee	356	1.77%	Maryland	Baltimore	200	0.99%
Indiana	355	1.76%	North Carolina	Charlotte	200	0.99%

Table 2 shows summary statistics for the median scores for GSV-derived built environment indicators (the percentage of images in a given census tract with the indicator of interest) by city, for those cities with ten or more census tracts. Street greenness scores ranged from 0.23 to 0.97, crosswalk scores from < 0.01 to 0.53, building type (not a single-family home) scores from 0.08 to 0.98, single-lane road scores from 0.09 to 0.80, and visible wire scores from 0.29 to 0.96. The states with the highest median census tract scores for street greenness were South Carolina, North Carolina, and Georgia. For crosswalks, the top states (after the District of Columbia, which had the highest median census tract score for this indicator) were New York, New Jersey, and California.

**Table 2:** Summary statistics for GSV-derived built environment indicator median scores by city

GSV-Derived Indicator	Minimum	Lower Quartile	Median	Upper Quartile	Maximum
Street Greenness	0.227	0.722	0.821	0.887	0.974
Crosswalk	0.002	0.072	0.111	0.196	0.528
Not Single-Family Home	0.076	0.224	0.318	0.455	0.984
Single-Lane Road	0.033	0.389	0.532	0.618	0.804
Visible Wires	0.287	0.574	0.668	0.777	0.961

We modeled associations between GSV-derived built environment indicators, demographic and socioeconomic covariates, and health outcomes and behaviors from the 500 cities data set. Table 3 displays the analysis results. Street greenness was associated with decreased prevalence of physical distress (for the third tertile only), mental distress, and binge drinking, but increased prevalence of obesity. Visible wires (a possible indicator of physical disorder) were associated with increased prevalence of all health-related behaviors and outcomes except for obesity, which showed a negative association (for the third tertile only). Building types other than single-family homes (an indicator of mixed land use) were associated with decreased prevalence of obesity, diabetes, and inactivity, but with increased levels of mental distress (for the second tertile only) and binge drinking (for the third tertile only). More single-lane roads (an indicator of less urban development) were associated with higher prevalence of obesity, diabetes, physical distress (for the third tertile only) and decreased prevalence of mental distress (for the second tertile only) and binge drinking.

**Table 3:** Built environment predictors of health-related behaviors and outcomes

Street Greenness							Crosswalk					
	Coef.	SE	p	95% CI			Coef.	SE	p	95% CI		
Obesity												
Tertile 2	0.797	0.095	< 0.001	0.611	0.983	*	1.318	0.225	< 0.001	0.876	1.760	*
Tertile 3	0.929	0.117	< 0.001	0.700	1.157	*	−0.813	0.219	< 0.001	−1.242	− 0.384	*
SEP	2.345	0.081	< 0.001	2.187	2.503		2.469	0.079	< 0.001	2.313	2.624	
Diabetes												
Tertile 2	0.002	0.038	0.961	−0.073	0.077		0.354	0.087	< 0.001	0.184	0.524	*
Tertile 3	0.009	0.046	0.852	−0.082	0.099		−0.174	0.085	0.040	−0.340	−0.008	*
SEP	1.665	0.036	< 0.001	1.594	1.736		1.705	0.036	< 0.001	1.634	1.776	
Physical Distress												
Tertile 2	−0.062	0.041	0.128	−0.142	0.018		0.507	0.096	< 0.001	0.320	0.695	*
Tertile 3	−0.143	0.049	0.004	−0.239	− 0.047	*	0.438	0.093	< 0.001	0.256	0.621	*
SEP	2.535	0.038	< 0.001	2.461	2.608		2.540	0.038	< 0.001	2.466	2.614	
Mental Distress												
Tertile 2	−0.075	0.038	0.047	− 0.149	− 0.001	*	0.499	0.090	< 0.001	0.323	0.676	*
Tertile 3	−0.124	0.046	0.007	−0.214	− 0.034	*	0.559	0.087	< 0.001	0.388	0.730	*
SEP	2.230	0.033	< 0.001	2.165	2.294		2.224	0.033	< 0.001	2.159	2.289	
Physical Inactivity												
Tertile 2	0.001	0.090	0.995	−0.177	0.178		0.502	0.191	0.009	0.127	0.877	*
Tertile 3	−0.019	0.106	0.860	−0.227	0.189		−1.124	0.188	< 0.001	−1.494	− 0.755	*
SEP	4.499	0.078	< 0.001	4.346	4.652		4.630	0.078	< 0.001	4.478	4.782	
Binge Drinking												
Tertile 2	−0.721	0.064	< 0.001	− 0.847	− 0.594	*	1.577	0.138	< 0.001	1.307	1.848	*
Tertile 3	−0.912	0.083	< 0.001	−1.074	− 0.749	*	3.005	0.133	< 0.001	2.743	3.266	*
SEP	−0.873	0.055	< 0.001	− 0.980	− 0.765		− 0.968	0.053	< 0.001	−1.073	− 0.864	
Not Single-Family Home							Single Lane Road					
	Coef.	SE	p	95% CI			Coef.	SE	p	95% CI		
Obesity												
Tertile 2	−0.753	0.125	< 0.001	−0.999	− 0.508	*	2.202	0.096	< 0.001	2.014	2.389	*
Tertile 3	−2.535	0.127	< 0.001	−2.783	−2.286	*	3.378	0.110	< 0.001	3.162	3.594	*
SEP	2.489	0.080	< 0.001	2.333	2.645		2.501	0.078	< 0.001	2.348	2.654	
Diabetes												
Tertile 2	−0.199	0.049	< 0.001	−0.295	−0.104	*	0.308	0.040	< 0.001	0.231	0.386	*
Tertile 3	−0.437	0.051	< 0.001	−0.537	− 0.338	*	0.722	0.045	< 0.001	0.634	0.810	*
SEP	1.694	0.036	< 0.001	1.623	1.766		1.703	0.036	< 0.001	1.632	1.774	
Physical Distress												
Tertile 2	0.001	0.052	0.979	−0.101	0.103		0.048	0.042	0.257	−0.035	0.131	
Tertile 3	−0.086	0.054	0.109	−0.191	0.019		0.269	0.048	< 0.001	0.175	0.362	*
SEP	2.546	0.037	< 0.001	2.473	2.620		2.552	0.037	< 0.001	2.479	2.625	
Mental Distress												
Tertile 2	0.110	0.049	0.026	0.013	0.206	*	−0.129	0.039	0.001	−0.206	−0.052	
Tertile 3	0.096	0.050	0.057	−0.003	0.195	§	−0.079	0.044	0.076	−0.165	0.008	§
SEP	2.229	0.033	< 0.001	2.165	2.294		2.228	0.033	< 0.001	2.163	2.292	
Physical Inactivity												
Tertile 2	−0.637	0.110	< 0.001	−0.853	− 0.421	*	0.767	0.095	< 0.001	0.581	0.953	
Tertile 3	−0.950	0.115	< 0.001	−1.175	−0.726	*	1.896	0.104	< 0.001	1.693	2.100	
SEP	4.558	0.078	< 0.001	4.405	4.711		4.599	0.078	< 0.001	4.447	4.751	
Binge Drinking												
Tertile 2	0.140	0.087	0.107	−0.030	0.311		−0.720	0.066	< 0.001	−0.850	− 0.590	*
Tertile 3	1.189	0.087	< 0.001	1.019	1.359	*	−0.901	0.075	< 0.001	−1.048	−0.754	*
SEP	−0.928	0.054	< 0.001	−1.034	−0.821		−0.890	0.054	< 0.001	−0.997	−0.784	
Visible Wires												
	Coef.	SE	p	95% CI								
Obesity												
Tertile 2	0.191	0.125	0.127	−0.054	0.436							
Tertile 3	−0.415	0.120	0.001	−0.651	−0.180	*						
SEP	2.385	0.082	< 0.001	2.226	2.545							
Diabetes												
Tertile 2	0.218	0.047	< 0.001	0.127	0.309	*						
Tertile 3	0.514	0.046	< 0.001	0.423	0.605	*						
SEP	1.585	0.037	< 0.001	1.512	1.657							
Physical Distress												
Tertile 2	0.272	0.051	< 0.001	0.173	0.371	*						
Tertile 3	0.738	0.050	< 0.001	0.641	0.836	*						
SEP	2.423	0.038	< 0.001	2.349	2.496							
Mental Distress												
Tertile 2	0.171	0.049	0.001	0.074	0.267	*						
Tertile 3	0.471	0.047	< 0.001	0.379	0.564	*						
SEP	2.159	0.033	< 0.001	2.094	2.224							
Physical Inactivity												
Tertile 2	0.688	0.108	< 0.001	0.478	0.899	*						
Tertile 3	1.001	0.109	< 0.001	0.788	1.214	*						
SEP	4.356	0.079	< 0.001	4.201	4.510							
Binge Drinking												
Tertile 2	0.487	0.090	< 0.001	0.310	0.664	*						
Tertile 3	0.732	0.084	< 0.001	0.567	0.896	*						
SEP	−0.941	0.056	< 0.001	−1.050	−0.831							

*p < 0.05

§*p* < 0.10

*SEP* Composite economic factor for socioeconomic position

Relationships with crosswalks were complex. Crosswalks (an indicator of walkability) exhibited a U-shaped relationship for obesity, diabetes and physical inactivity. Areas with the most crosswalks (third tertile) experienced a reduction in obesity, diabetes and physical activity. However, the second tertile experienced higher rates of obesity, diabetes and physical activity compared to the first (lowest) tertile. Additionally, crosswalks were associated with higher prevalence of both physical and mental distress, as well as binge drinking.

Socioeconomic disadvantage was negatively associated with binge drinking prevalence and positively associated with all other health-related behaviors and outcomes. When examining demographic characteristics (data shown in Additional file 1: Table S1), census tracts with a higher proportion of women was associated with decreased prevalence of all behaviors and outcomes except for binge drinking, with which there was no association. A higher proportion of African American residents was associated with increased prevalence of obesity, diabetes, and inactivity, and with decreased prevalence of mental distress and binge drinking.

## Discussion

Structural determinants, including the built environment, can influence the health outcomes and behaviors of the populations that live among them. This analysis modeled the association between health outcomes and built environment indicators derived from Google Street View images for urban and suburban neighborhoods, given the composition of the 500 Cities Project data. Our use of GSV-derived indicators of built environment features contributes to a growing body of work that has focused on developing a wide variety of methods to measure these associations, particularly in urban areas [34–38]. These include GIS-measured street intersection density [34, 36, 38], residential density, land-use mix [38], and counts, population ratios, and densities of features of interest, including parks, intersections, subway stations, and green spaces [35, 37]. These analyses have found similar results to ours regarding both poverty and built environment features and health-related behaviors that affect obesity. For example, previous analyses have found inverse associations between neighborhood walkability and sedentary behavior [34, 38], obesity [35], diabetes, and hypertension [36]. Associations have also been observed between socioeconomic disadvantage and increases in adverse health outcomes such as sedentary behavior [38] and poor hypertension control [36].

We found that single lane roads, which may indicate lower levels of urban development (suburban areas) which structures fewer amenities where people live, were associated with increased diabetes and obesity. This is consistent with some of our prior work utilizing GSV images, where we found that indicators of greater urban development, such as crosswalks and mixed residential use, are associated with decreases in many adverse health outcomes, but slight increases in distress and binge drinking. For example, previous work using Google’s computer vision API to automatically label Google Street View images found that areas characterized as rural (limited infrastructure) had higher obesity, diabetes, fair/poor self-rated health, premature mortality, physical distress, physical inactivity and teen birth rates but lower rates of excessive drinking [14]. Similarly, we also found that non-single-family home buildings (an indicator of having a mixture of residential and commercial buildings nearby and thus dense offerings of services and amenities) were associated with decreased obesity, diabetes and inactivity.

**Fig. 1:**
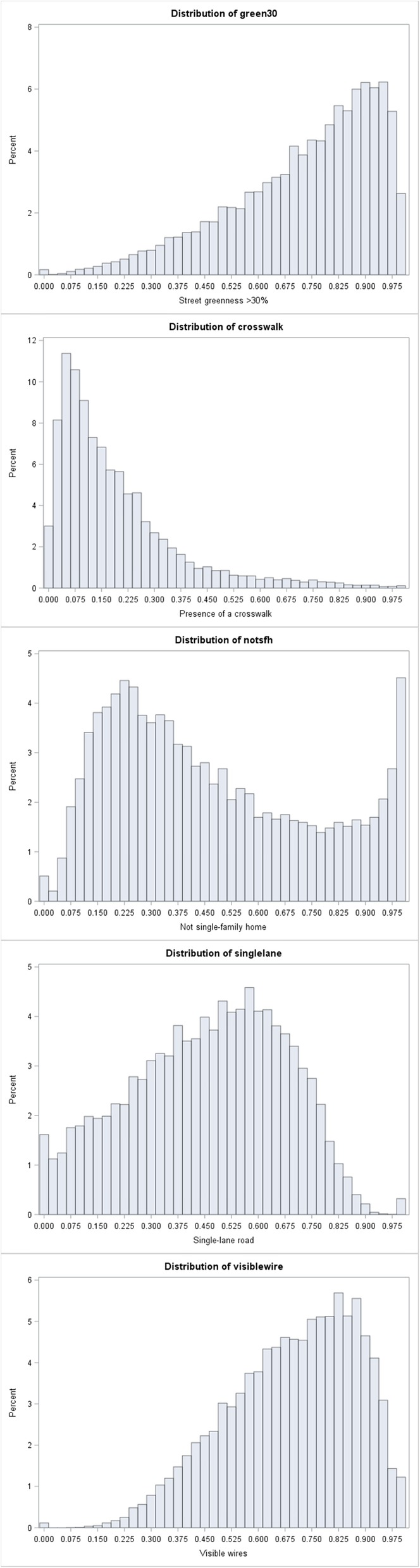
Distribution of built environment indicators

We observed a complex relationship between crosswalk score tertiles and obesity, diabetes, and inactivity, with the second tertile associated with an increased prevalence of these outcomes while the third tertile was associated with decreased prevalence. This relationship was observed in the univariate model (which were fit with only the crosswalk indicator and the outcome; data not shown) and persisted after adding covariates for demographic factors (data not shown) and socioeconomic disadvantage (Table 3) for all three outcomes. However, when we fit the same models using the crosswalk indicator as a linear variable, the indicator was negatively associated with all three outcomes (obesity − 7.37, 95% CI − 7.75 to − 7.00; diabetes − 0.91, 95% CI − 1.08 to − 0.73; inactivity − 0.92, 95% CI − 1.36 to − 0.49). The crosswalk indicator was substantially more right-skewed than any of the other GSV-derived indicators, so this relationship observed between tertiles may be a function of the unique distribution of this variable (Fig. 1).

The presence of plants has been tied to lower perceived stress and mental health [25]. We saw similar trends in our analysis, with street greenness associated with decreased prevalence of physical and mental distress, as well as decreased binge drinking. However, it was also associated with increased obesity. This may be due to the living situations and family structures of those living in greener areas. For example, if these are more suburban areas with larger proportions of families with children, the residents may be more sedentary than those who live in denser areas with more single people and greater levels of mixed land use. This could be supported by other associations observed, such as the associations seen between non-single-family home building types and decreased prevalence of obesity, diabetes, and inactivity; the relationship between single-lane roads, which are more prevalent in suburban areas, with increased prevalence of diabetes and obesity; and the association between crosswalks and increased binge drinking prevalence (and, similarly, the association between single-lane roads and decreased prevalence of binge drinking).

### Study strengths and limitations

Characterizing features of the built environment in the past has been time-consuming and cumbersome, typically requiring researchers to rely on self-report data from residents in neighborhood surveys or to conduct in-person audits that require auditors to physically record and detail the locations and features of indicators of interest for the desired geographic area. Our analysis expands on recent technological advances in computer vision and deep learning tools to create indicators for a high volume of images, allowing us to expand on previous work assessing health outcomes in relation to the built environment [14, 15]. To our knowledge, this is the first study examining these associations at the census tract level for a large number of U.S. cities.

This study is subject to several limitations. First, the analysis is an ecological one, as all of the data used to measure associations were aggregated. Thus, while the results may be used to inform policies or programs designed to address health-related outcomes at the population level (since that is the level at which the outcomes were measured), they should not be applied to programs to address individual behaviors or health outcomes. This gap can be addressed by work linking built environment indicators to individual health data, such as the work done by Le-Scherban et al. [36], which will allow associations to be measured while controlling for individual-level covariates. Second, the census tracts included in the analysis were limited to those with health-related behavior and outcome data from the 500 Cities Project, and so can only be generalized to urban areas in the U.S. Previous studies have shown major disparities in health outcomes between residents of urban versus rural areas [39]. Additional work is needed to better understand how built environment indicators may impact the health of residents of rural neighborhoods.

Third, there are also limitations inherent with the methods used to construct the built environment indicators. Because GSV images are taken at intersections, they cannot capture all information on the indicators of interest. Finally, images do not capture all of the features of the neighborhood environment that may impact health outcomes, such as traffic congestion and perceived safety, nor do they allow us to assess how the built environment changes over time. Additional sources of data should be identified that provide this information.

## Conclusions

The impact of the neighborhood’s built environment features on the people who live in it has been a focus of both neighborhood residents and social epidemiologists and, more recently, policy makers and advocates. Accompanying this interest is a growing interest in novel technological methods to characterize and measure these associations. Our analysis of the impact of built environment indicators on health outcomes and behaviors in cities, where 81% of Americans live [40], suggests that higher levels of urban development, such as mixed land use, multi-lane roads, crosswalks, and less greenery, have mixed effects on health, showing decreases in some adverse outcomes such as obesity, diabetes, and physical inactivity, with increases in others such as physical and mental distress and binge drinking. Visible wires were used as an indicator of physical disorder and were connected with higher prevalence of diabetes, physical and mental distress, physical inactivity, and binge drinking. Additionally, our results add further evidence that socioeconomic distress has adverse impacts on multiple physical and mental health outcomes. These insights on economic inequality and the built environment can be used by public health officials, advocates, and policy makers to inform work to address these structural factors that impact public health.

## Supplementary information

**Additional file 1.** Built environment predictors of health-related behaviors and outcomes, with full regression results for demographic covariates.

## Data Availability

The dataset(s) supporting the conclusions of this article is (are) available in the Open ICPSR repository, 10.3886/E115264V1.

## Abbreviations

ACS: American Community Survey
API: Application programming interface
BRFSS: Behavioral Risk Factor Surveillance System
CDC: Centers for Disease Control and Prevention
GSV: Google Street View
TIGER: Topologically Integrated Geographic Encoding and Referencing
